# The possible positive effects of physical exercise on the global motion perception aging: the cognitive mechanism

**DOI:** 10.3389/fpsyg.2024.1323291

**Published:** 2024-01-24

**Authors:** Ziping Liang, Lei Zhang, Pengpeng Wang, Yuping Zhang, Yaoyuan Xia, Hua Jin

**Affiliations:** ^1^Mental Health Education Center, Zhengzhou University, Zhengzhou, Henan, China; ^2^School of Physical Education (Main Campus), Zhengzhou University, Zhengzhou, Henan, China; ^3^College of Psychology, Xinjiang Normal University, Urumqi, China; ^4^Medicine School of Rehabilitation, Henan University of Chinese Medicine, Zhengzhou, Henan, China; ^5^Department of Physical Education, Zhejiang University of Finance and Economics, Hangzhou, Zhejiang, China; ^6^Key Research Base of Humanities and Social Sciences of the Ministry of Education, Academy of Psychology and Behavior, Faculty of Psychology, Tianjin Social Science Laboratory of Students’ Mental Development and Learning, Tianjin Normal University, Tianjin, China

**Keywords:** exercise, global motion perception, aging, perceptual template model, cognitive

## Abstract

**Background:**

Sensitivity to global motion perception (GMP) decreases gradually with age, and the mechanism to effectively alleviate its aging process is still unclear. This study aimed to examine the impact and mechanism of exercise on GMP aging.

**Methods:**

This study adopted the global motion direction discrimination task and used motion coherence thresholds to assess GMP sensitivity. It adopted the perceptual template model (PTM) to fit the GMP processing efficiency.

**Results:**

The threshold for the elderly group with no exercise was higher than that of the elderly group with exercise, while the threshold of the latter was higher than that of the youth group. The results of the model fitting showed that both models, A_*a*_ and A_*f*_, corresponding to the elderly group with exercise and the elderly group with no exercise, respectively, were the best-fitted models when compared with that of the youth group. Compared to the elderly group with no exercise, models A_*a*_ and A_*f*_, were the best-fitted models.

**Conclusion:**

These results showed that good exercise habits might have a certain degree of positive effect on GMP aging, by lower their internal additive noise (A_*a*_), and improve the ability to eliminate external noise (A_*f*_).

## 1 Introduction

Global motion perception (GMP) is an important component of visual perception, and it refers to the selective integration of individual trajectories of local motion elements in the visual scenes based on certain rules, which form the motion perception of the entire scene (Hutchinson et al., 2012; Cai et al., 2014). It consists mainly of horizontal and vertical translational flows, as well as rotational and radial complex optical motion flows (Hutchinson et al., 2012). Studies show that GMP shows an aging tendency and manifests itself in the form of both lowered global motion sensitivity (GMS) and perceptual efficiency of the elderly (Hutchinson et al., 2012; Billino and Pilz, 2019). It is also influenced by the impact of factors, such as speed (Ward et al., 2018) and contrast (Allen et al., 2010). For example, Ward et al. (2018) recruited 28 young, 22 middle-aged and 23 older adults to identify indicators of perceptual decline in motion processing. They found that the value of individual Motion Coherence Threshold (MTC) showed a robust Pearson’s skipped correlation of age associated with poorer motion perception at 3 and 9°/s stimulus speeds. GMP aging has a severe impact on the interaction of the elderly and their dynamic surroundings and affects the quality of their lives, as well as their safety. Hence, in view of the increasingly aging society, it is necessary to identify a mechanism that can alleviate GMS aging and decline perceptual efficiency of global motion information for the elderly.

Psychologists have primarily adopted an external noise method to determine the mechanism for perceptual efficiency changes, and the perceptual template model (PTM) has been widely applied in this field (Lu and Dosher, 1999). This model measures the signal stimulus energy required of an observer to achieve a given level of performance as a function of external noise by systematically manipulating the intensity of external noise applied to the signal stimulus. The model identifies the following three mechanisms that characterize perceptual improvements: lowering of internal additive noise, improving the ability to eliminate external noise, and lowering of multiplicative internal noise. For example, Bejjanki et al.’s (2014) study on a fitted PTM revealed that the enhanced perceptual learning ability in action video game players has benefited from a combination of mechanisms to improve the ability to eliminate external noise and reduce internal additive noise. Bower and Andersen (2012) calculated the minimum contrast between the signal point and the noise point required for the participants to judge the global motion information of the random lattices, including noise changes, and discovered that the contrast threshold for the elderly group increased significantly compared to that of the youth group, and this was manifested in the reduction of perceptual efficiency in processing global signals. The results of the fitted PTM showed that the reduction in perceptual efficiency was mainly caused by the enhancement of its internal additive noise and the weakening of its ability to eliminate external noise. There were some improvements to the two noise mechanisms of the elderly when they were given laboratory perception training together with the youth group. The degree of reduction in internal additive noise for the elderly group was lower than that of the youth group, and the enhancement ability of the elderly group to exclude external noise was higher than that of the youth group. However, these studies adopted mainly static contrast for external noise, and none of the studies applied the GMP processing mechanism, whereby GMP aging is also affected by dynamic velocity noise.

The above studies have also shown that the GMP of the elderly is still quite malleable and can be improved through laboratory perception training (Ball and Sekuler, 1986; Bower and Andersen, 2012). However, the perception training adopted in these studies was realized in the laboratories, and this kind of improvement is very specific and is rarely transferrable between experimental tasks with low homogeneity. The strong specificity of this kind of learning effect lacks serious ecological validity, thus restricting its application in real life. Additionally, it is very difficult for most professionals to face the same tasks and conditions, such as laboratory perception training, as very few professional practitioners could face in real life. In addition, a unique life training experience can also improve the GMP sensitivity of an individual to a certain extent (Overney et al., 2008; Green et al., 2010; Kong et al., 2012; Hutchinson and Stocks, 2013; Pavan et al., 2016; Jin et al., 2019). For example, Pavan et al. (2016) found that the GMS of action video game players were significantly lower than those of non-action video game players and non-video game players. Similarly, a study by Jin et al. (2019) revealed a significantly higher GMS in badminton players compared with non-sportsmen, and the fitted PTM results showed that badminton players had stronger external noise elimination abilities. However, all bodies of evidence are from experiments targeting young people, whereas the elderly are unable to complete such vigorous activities and action video games, which require the operation of electronic products due to physical and lifestyle conditions, etc. Instead, most of the elderly perform their daily exercises during their free time by participating in prescribed elderly activities such as walking, parade dances, and the use of gym equipment (Yang et al., 2019). Studies have shown that long-term exercise can improve the cardiorespiratory fitness of the elderly and improve the flexibility and mental agility of the central nervous system (Liang, 2001; Lautenschlager et al., 2008; Ngandu et al., 2015), which can effectively promote neurogenesis and neuroplasticity, and delay cranial nerve aging (Paillard et al., 2015; Radak et al., 2016). Meanwhile, Witte et al. (2016) found that 10 5-month karate training could help to enhance cognitive function, such as attention, resilience, and motor reaction time, but a training period of 10 months was even more efficient. Godde and Voelcker-Rehage (2017) found that walking and coordination exercises reduced right dorsolateral prefrontal cortex activation. Muiños and Ballesteros (2018) summarized a review about the improvement of physical exercise on the perceptual skills and visuospatial attention in older adults. The review showed that research had demonstrated that physical activity might act as a strategy and be one of the main factors that could slow down age-related perceptual and cognitive decline. These results suggested a relationship between physical exercise and the cognitive functions, including perception. Vaina et al. (2001) also found that the status of one’s brain health (cerebral infarction) will also impact its GMS. Hence, it is highly practical to study how daily exercise leads to improved GMP aging of the elderly and provide a specific and feasible theoretical basis for alleviating GMP aging.

Hence, this study adopted the classical random-dot kinematogram paradigm. First, two kinds of translational motions–horizontal and vertical, and two kinds of complex optical motion flow–rotational and radial, were used to investigate the presence of GMP aging in the elderly group with exercise and the elderly group with no exercise under these four forms of exercise (Experiment 1). Afterward, we compared GMP difference between the elderly group with exercise and the elderly group with no exercise by adopting dynamic speed as external noise using the PTM technique. We also investigated the perceptual mechanism and its differences when processing global motion signals by comparing the youth control group, elderly control group with exercise, and elderly control group with no exercise (Experiment 2). Based on previous studies, we assumed the following: (1) maintaining good exercise habits may alleviate GMP decline in the elderly to a certain extent, (2) the impact of exercise on the GMP processing efficiency may be caused by the effect of one or more mechanisms of internal additive noise, the ability to eliminate external noise, and multiplicative internal noise.

## 2 Experiment 1

We used the random-dot kinematograms of the global motion direction discrimination task, in the horizontal, vertical, rotational, and radial directions, and compared the behavior performance of the youth group, the elderly group with exercise, and the elderly group with no exercise, in completing these four tasks. Thereafter, we studied the impact of exercise on alleviating GMP aging.

### 2.1 Research method

#### 2.1.1 Design

We adopted a two-factor mixed experimental design. The groups were between-subjects variables with the youth group as a control group, and the elderly group divided into the exercise and no-exercise groups. The motion direction was the within-subjects variable, and it was divided into horizontal leftward and rightward, vertical upward, and downward, clockwise and anticlockwise rotation, and radial contraction and expansion. The dependent variable was the motion coherence threshold (“threshold” in short), which was the proportion of minimum signal points required by an individual to judge the global motion direction of the lattice to the total number of points. The higher the threshold value, the lower the GMS and perceptual efficiency, and vice versa.

#### 2.1.2 Participants

G*Power software calculated that at least 10 subjects were required for each group in this study (Effect size *f* = 0.25, α = 0.05, 1-β = 0.95). In this experiment, we used the Acuity E and Contrast C tests in the FrACT 3.9.8 software (Freiburg visual acuity test, michaelbach.de/fract/) to eliminate individuals with vision or corrected vision < 0.8, and those with contrast visual acuity < 1.35, respectively (Bach, 1996). We used the mini-mental state examination (MMSE) to assess the cognitive state of the elderly between-subjects and eliminated individuals with MMSE scores < 24 (Folstein et al., 1975; Porter et al., 2017). Finally, 21 youths (11 males, 19.99 to 26.33 years, average age of 22.45 ± 1.88 years), 18 elderly with exercise habits (9 males, 61.27 to 71.11 years, average age of 67.11 ± 2.94 years, average MMSE score of 22.67 ± 1.94), and 18 elderly with no exercise habits (6 males, 62.36 to 70.61 years, average age of 65.34 ± 2.79 years, average MMSE score of 22.78 ± 1.66), were included in the final data analysis. The age differences between the elderly group with exercise and the elderly group with no exercise were not significant [*t*(34) = –1.86, *p* = 0.07], and the difference in MMSE scores was also not significant [*t*(34) = 0.18, *p* = 0.86].

The criteria for inclusion of the elderly as an exercise group was based on the following: (1) active and regular participation in exercises such as slow or brisk walking, using gym equipment or taking part in group exercise activities such as Tai Chi, for at least a year, apart from daily routine activities, (2) average daily exercise time of 1 to 2 h, and (3) minimum average weekly exercise time of 7 h. The criteria for inclusion in the youth group and the elderly with no exercise group was based on the following criterion: (1) no regular exercise habits within the past 1 year apart from essential activities such as go to the classroom, routine shopping or fetching of young children, (2) average daily exercise time shorter than 0.5 h, and (3) average weekly exercise time shorter than 3 h.

All participants had normal vision or corrected vision and had no amblyopia, cataract, glaucoma, macular degeneration, astigmatism, or other vision disorders, no addiction to cigarettes and alcohol. They also had no cognitive impairment, or intellectual disability due to various reasons, no mental illnesses such as schizophrenia, and no neurosis such as depression and obsessive-compulsive disorders. All participants were compensated for their participation in the study and had signed informed consent agreements before the experiment.

#### 2.1.3 Materials

**FIGURE 1:**
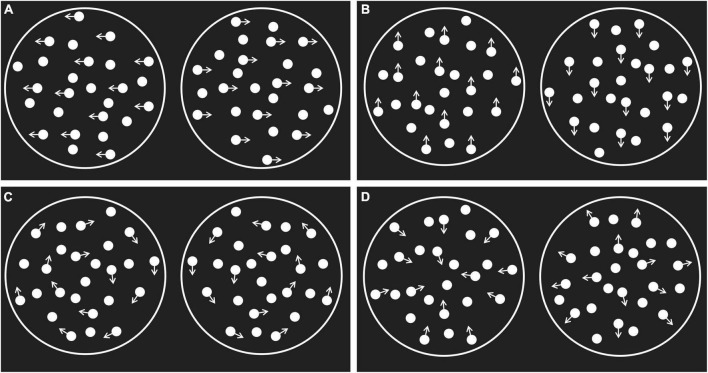
The random dot kinematogram stimuli. **(A)** The horizontal motion; **(B)** the vertical motion; **(C)** the rotational motion; **(D)** the radial motion. The arrow indicates the direction of the movement of the signal dot.

The stimuli were the horizontal, vertical, rotational, and radial random-dot kinematograms (RDKs) presented by the Psych Toolbox in the MATLAB software^1^ (Brainard, 1997; Pelli, 1997). The research materials were similar to the ones used by Liang et al. (2021) screen refresh rate of 60 Hz, resolution of 1,920 × 1,080 pixels, width of 38.2 cm, and height of 21.5 cm. The lattices were presented inside a white circular aperture in the middle of the black screen (radius of 6 cm, sight angle of 11.42°), with a sight distance of 60 cm. There were 1,000 white dots in the lattices (density of 8.85 dot/cm^2^, size of 2 pixels), and the movement speed of all dots was 5°/s, with a duration of 50 ms. The direction of some dots in the lattices was the same as the signal points, while the movement direction of the remaining dots used as noise points was random. The movement direction of the signal points is shown in Figure 1.

#### 2.1.4 Program and tasks

**FIGURE 2:**
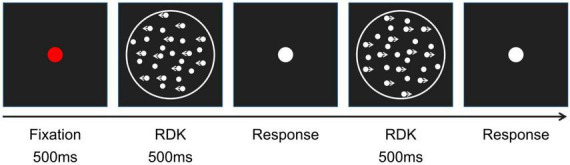
Global motion direction discrimination experimental procedure.

The experiment was carried out in a quiet room, and a 500 ms red gaze point was presented in the middle of the screen after the procedure had started, to remind the participants that the experiment had begun. After that, the screen displayed the 500 ms lattices, followed by the white dots. Participants were requested to point out the overall motion direction of the lattices by operating the buttons as accurately as possible (we did not request speed in their answers) after the lattice stimuli had appeared. To prevent the program from freezing, the white dots continued for another 200 ms after participants had provided their responses (Figure 2). Before the experiment, participants were told that the signal points would move randomly in two opposite directions with equal occurrence probability for each of the motion direction tasks, and they were asked to make their best judgment under uncertain circumstances (Liang et al., 2021).

The experiment consisted of four blocks, corresponding to the four motion forms of horizontal, vertical, rotational, and radial, and the presentation order of the blocks was balanced across participants. Each block consisted of six sessions, of which the first session was used as a trial and not included in the final analysis. Using the “3 down 1 up” adaptive staircase to manipulate the motion coherence level of the stimuli, the consistency level would decrease by one step if all the reaction responses of the participants were accurate and consistent for three consecutive times, and it would be increased by one step if they had made one reaction error. Thus, we used this to track the threshold at 79.37% accuracy (Lu and Dosher, 1999). The proportion of signal points moving consistently in each trial varied with the responses of the participants, and they would be asked to complete eight reversals in any of the two motion directions appearing randomly. The starting consistency level of the lattices was 100%, with a step size of 10% for the first two times, and 5% for the last six times. We separately calculated the average consistency levels of the last six reversals at the turning point with a step size of 5% for each motion direction as the threshold of the individual in this direction and obtained the threshold of this motion form by averaging the threshold levels of the two motion directions (Allen et al., 2010; Hutchinson et al., 2014).

#### 2.1.5 Data analysis

Taking ± 3 standard deviations of the average values of data from the youth group, elderly group with exercise, and elderly group with no exercise as the upper and lower limits for conducting outlier removal, no outlier data was found in the these groups. All data from the participants were included in subsequent data analysis, and the IBM SPSS 25.0 software was used for processing the data.

First, the threshold values for the youth group, elderly group with exercise, and elderly group with no exercise, were analyzed in terms of 3 (groups) × 4 (motion forms) using the Two-Way Repeated-Measures ANOVA, and the differences between motion forms were obtained. Next, the threshold values were analyzed in terms of 3 (groups) × 8 (exercise directions) using the Two-Way Repeated-Measures ANOVA, and the differences between motion directions were obtained.

### 2.2 Result

#### 2.2.1 Differences in motion coherence threshold values of four motion forms

Comparison of the threshold values of the three groups under the four forms showed that (Table 1) the main effect of the group was significant [*F*(2,54) = 71.66, *p* < 0.001, η_*P*_^2^ = 0.73], with the threshold of the elderly group with no exercise being significantly higher than that of the elderly group with exercise (*p* < 0.001), and the threshold of the latter being higher than that of the youth group (*p* < 0.001). The main effect of the motion form was significant [*F*(3,162) = 14.10, *p* < 0.001, η_*P*_^2^ = 0.21]. The interaction between the groups and motion forms was significant [*F*(6,162) = 4.22, *p* = 0.001, η_*P*_^2^ = 0.14].

**TABLE 1:** The motion coherence threshold in 4 motion forms of the youth and the elderly groups (%, M ± SD).

	Horizontal	Vertical	Rotational	Radial
Youth	25.25 ± 7.22	29.71 ± 8.46	29.05 ± 8.01	18.54 ± 4.92
Elderly with exercise	40.88 ± 17.72	45.54 ± 16.02	38.61 ± 13.58	38.50 ± 19.42
Elderly with no exercise	72.62 ± 17.09	79.32 ± 14.77	68.72 ± 18.12	73.23 ± 15.93

The main purpose of this study was to compare the differences in threshold values among different motion forms of the three groups. Thus, we defined the motion form as the fixed factor during the course of conducting *post-hoc* tests of the interaction effect. Our results have shown that the threshold value of the elderly group with no exercise was significantly higher than that of the elderly group with exercise for the radial, horizontal, and vertical motion forms (*ps* < 0.01), and the threshold value of the latter was higher than that of the youth group (*p* < 0.01). The threshold value of the elderly group with no exercise was significantly higher than that of the elderly group with exercise for the rotational motion form (*p* < 0.01), but the difference between the latter and the youth group was not significant (*p* < 0.099).

#### 2.2.2 Differences in motion coherence threshold values of eight motion directions

**FIGURE 3:**
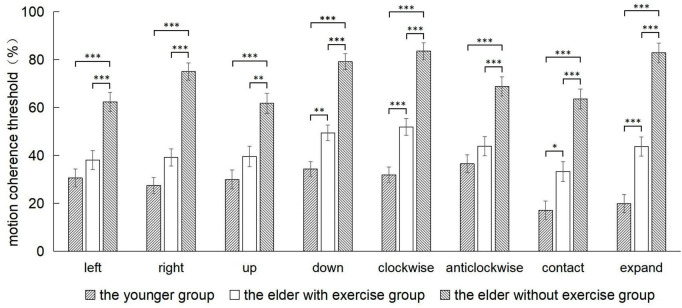
The comparison of the motion coherence threshold among the 3 groups in 8 directions. *p*-values in *post-hoc* analyses of one-way ANOVA: *p < 0.05, **p < 0.01, ***p < 0.001.

The comparison of threshold values among three groups of participants in eight motion directions showed that (Figure 3; Table 2) the main group effect was significant [*F*(2,54) = 22.75, *p* < 0.001, η_*P*_^2^ = 0.71], and the threshold value of the elderly group with no exercise was significantly higher than that of the elderly group with exercise (*p* < 0.001), and the threshold value of the groups were both higher than that of the youth group (elderly group with exercise *p* = 0.001, elderly group with no exercise *p* < 0.001). The main effect of motion direction was significant [*F*(7,378) = 13.35, *p* < 0.001, η_*P*_^2^ = 0.20], while the interaction between the group and motion direction was also significant [*F*(14,378) = 4.30, *p* < 0.001, η_*P*_^2^ = 0.14].

**TABLE 2:** The motion coherence threshold in 4 motion forms and 8 motion directions of the youth and the elderly groups (%, M ± SD).

	Horizontal		Vertical		Rotational		Radial	
	Left	Right	Up	Down	Clockwise	Anticlock-wise	Contract	Expand
Youth	30.60 ± 9.90	27.49 ± 10.69	29.98 ± 9.81	34.39 ± 9.78	31.93 ± 10.82	36.52 ± 11.45	17.17 ± 7.57	19.91 ± 7.28
Elderly with exercise	38.04 ± 16.24	39.18 ± 15.72	39.55 ± 16.21	49.44 ± 16.70	51.91 ± 19.97	43.90 ± 19.32	33.28 ± 19.77	43.72 ± 25.13
Elderly with no exercise	62.37 ± 23.50	75.07 ± 18.93	61.73 ± 25.74	79.13 ± 15.04	83.57 ± 12.86	68.88 ± 19.82	63.58 ± 22.63	82.87 ± 15.79

We defined the motion direction as the fixed factor during the course of conducting *post-hoc* tests of the interaction effect. Our results have shown that, the threshold value of the elderly group with exercise was significantly lower than that of the elderly group with no exercise in the leftward (*p* < 0.001), rightward (*p* < 0.001), upward (*p* = 0.002), downward (*p* < 0.001), clockwise (*p* < 0.001), anticlockwise (*p* < 0.001), contraction (*p* < 0.001), and expansion (*p* < 0.001) directions. And there was no significant difference in threshold values between the elderly group with exercise and the youth group in leftward (*p* < 0.546), rightward (*p* = 0.062), upward (*p* = 0.316), and anticlockwise (*p* = 0.548) directions.

## 3 Experiment 2

From the results of Experiment 1, the performance of the elderly group with exercise was overall better than that of the elderly group with no exercise across the four tasks in eight directions. In addition, their performance was similar to that of the youth group under conditions of horizontally leftward and rightward, vertically upward, and anticlockwise, directions. This showed that exercise can effectively and universally effect aging GMP in the elderly. However, the effect mechanism is still unclear. In Experiment 2, we used the PTM technique to divide the external speed noise fitted by the PTM into 10 levels and investigated the perceptual mechanism and improvement mechanism of exercise on alleviating aging GMP.

### 3.1 Research method

#### 3.1.1 Design

We adopted a three-factor mixed experimental design. The groups were between-subjects variables, which were divided into the youth group, the elderly group with exercise, and the elderly group with no exercise. The behavior level and speed noise were the within-subjects variables, and the behavior level was divided into 70.71% and 79.37% accuracy levels, and the speed noise was divided into 1°/s, 1.4°/s, 1.8°/s, 2.4°/s, 3°/s, 3.8°/s, 4.8°/s, 6°/s, 7.5°/s, and 9.5°/s. The dependent variable was the threshold.

#### 3.1.2 Participants

G*Power software calculated that at least 6 subjects were required for each group in this study (Effect size *f* = 0.25, α = 0.05, 1-β = 0.95). We asked for volunteers from the three groups in Experiment 1 to take part in this experiment, and 12 persons from each group were eventually designated as qualified participants: youth group (6 males, 20.67 to 23.20 years, average age of 21.94 ± 1.81 years), elderly group with exercise (3 males, 66.65–69.18 years, average age of 67.91 ± 2.36 years, average MMSE score of 22.17 ± 1.47), and the elderly group with no exercise (4 males, 63.17–65.71 years, average age of 64.44 ± 2.26 years, average MMSE score of 22.92 ± 1.08). The age of the elderly group with exercise was significantly higher than that of the elderly group with no exercise [*t*(22) = −3.68, *p* = 0.001, Cohen’s *d* = 1.50], but the difference in MMSE scores between the two groups was not significant [*t*(22) = −0.48, *p* = 0.640].

#### 3.1.3 Materials

The stimuli were the horizontal random-dot kinematograms (RDKs), and the presentation machine, aperture, number, density, and size of the dots, in the random lattices were the same as in Experiment 1. The external noise was achieved by changing the motion speed of the signal point, and 10 speed levels were determined according to the rules of the fitted PTM rules and pre-experiments: 1°/s, 1.4°/s, 1.8°/s, 2.4°/s, 3°/s, 3.8°/s, 4.8°/s, 6°/s, 7.5°/s, and 9.5°/s. The motion speed of the noise and signal points was consistent.

#### 3.1.4 Program and tasks

The experiment was carried out in a quiet room, and the presentation sequence and duration of the stimuli in each trial were similar to that of Experiment 1. The participants were requested to point out the overall motion direction of the lattices by operating the buttons (leftward: press F; rightward: press J) as accurately as possible (we did not request speed in their answers) after the lattice stimuli had appeared. Using the “2 down 1 up” and “3 down 1 up” adaptive staircases to manipulate the motion coherence level, we tracked the threshold behavior levels at 70.71% accuracy (*d*’ = 1.089) and 79.37% accuracy (*d*’ = 1.634), respectively. The initial consistency level of the lattices, the number of reversals, and the step size of the reversals were the same as in Experiment 1, and the average value of the last six reversals was taken to be the threshold of the participants (Lu and Dosher, 1999).

The experiment consisted of two sessions, with around 1,200 trials in each session, which was divided into 20 blocks. Each block only corresponded to one behavior level and speed noise. 20 training trials were conducted before the actual experiment, and the entire experiment lasted for 50 min, with the participants determining their own break time between blocks based on their health conditions.

#### 3.1.5 Data analysis

(1) Differences in motion coherence threshold values. Three factors of repeated measurement ANOVA were performed for the threshold values under the two behavior levels and 10 speed noises in three groups.

(2) The processing mechanism of the aging GMP. After fitting the threshold values of the three groups using the PTM technique and the Curve Fitting Toolbox in the MATLAB R2012a software, the model fitting procedures, and formula parameters are provided below (Lu and Dosher, 1999, 2004; Xu et al., 2006).

(a) Using the youth group data as the benchmark, we set *A*_*a*_= *A*_*f*_= *A*_*m*_= 1 into Formula 1 to fit the parameters of β, γ, N_*m*_, and N_*a*_, and obtained the optimal model for the youth group. (b) Setting the values of β, γ, N_*m*_, and N_*a*_, obtained in the last step into Formula 1 to fit the values of *A*_*a*_, *A*_*f*_, and *A*_*m*_ of the elderly group with exercise and the elderly group with no exercise at the two behavior levels, respectively. From there, we obtained the whole-parameter model with all three types of noises, the concise models with one or two parameters, and a parameter-free model. (c) Calculated the degree of fitting (*r*^2^) for each model (Formula 2). To obtain the best model, *F*-Test (Formula 3) was used to compare the fit degree of all models in the elderly group with exercise and the elderly group with no exercise. (d) To determine changes in the multiplicative internal noise, the paired-sample *t*-tests were conducted for the threshold ratio of the youth group/elderly group with exercise, and the youth group/elderly group with no exercise at the two behavior levels, respectively. The lack of significance indicates no change (Lu and Dosher, 1999; Bejjanki et al., 2014).


Formula⁢1:Cτ=1β⁢[(1+Am2⁢Nm2)⁢(Af2⁢γ⁢Ne⁢x⁢t2⁢γ)+Aa2⁢Na21/d′2-Am2⁢Nm2]12⁢γ

$$\mathrm{Formula}\,2:r^{2}=1.0-\frac{\sum {\left(\log\left(c^{t\,h\,e\,o\,r\,y}\right)-\log\left(c\right)\right)}^{2}}{\sum {\left(\log\left(c\right)-m\,e\,a\,n\,\left(\log\left(c\right)\right)\right)}^{2}}$$

$$\mathrm{Formula}\,3:F\,\left(d\,f_{1},d\,f_{2}\right)=\frac{\left(r_{f\,u\,l\,l}^{2}-r_{r\,e\,d\,u\,c\,e\,d}^{2}\right)/d\,f_{1}}{\left(1-r_{f\,u\,l\,l}^{2}\right)/d\,f_{2}}$$

Formula 1: Perceptual Template Model. c_τ_ is the threshold required to maintain a designated behavior level under designated external noise (*N*_*ext*_) conditions. *A*_*a*_, *A*_*f*_, and *A*_*m*_ reflected the changes in the internal additive noise (*N*_*a*_), the multiplicative internal noise (*N*_*m*_), and the ability to eliminate external noise (*N*_*ext*_), respectively. β is the gain control, γ is the non-linear portion of the model.

Formula 2: Fit. Σ and *mean*() included all behavior levels and external noise conditions. log(*c*_*theory*_) is the logarithm of the theoretical threshold for each model (this is obtained by setting the parameters obtained from each fitted model into Formula 1) and log(*c*) is the logarithm of the actual measure threshold.

Formula 3: *F*-test. *df*_1 _= *k*_*full*_-*k*_*reduced*,_ *df*_2 _= *N*-*k*_*reduced*_. *k* is the number of parameters in each model, and *N* is the number of predicted data points [that is, 2 (groups) × 2 (behavior levels) × 10 (noise levels) = 40].

(3) Impact of exercise on the mechanism of aging GMP. Using the elderly group with no exercise as the benchmark and the above-mentioned PTM as the fitting step, we obtained the optimal fitted model for processing global motion information of the elderly group with exercise.

### 3.2 Result

#### 3.2.1 Differences in motion coherence threshold values of three groups of participants

When comparing the threshold values of the three groups of participants, the results showed that (Table 3) the main group effect was significant [*F*(1,33) = 16.08, *p* < 0.001, η_*p*_^2^ = 0.49] and the threshold value of the elderly group with no exercise was significantly higher than that of the elderly group with exercise (*p* = 0.008), with the latter group having a significant highly threshold value than the youth group (*p* = 0.007). The main effect of behavior level was significant [*F*(1,33) = 91.91, *p* < 0.001, η_*p*_^2^ = 0.74], while the threshold value at 70.71% accuracy was significantly lower than that at 79.37% (*p* < 0.001). The main effect of motion speed was significant [*F*(9,297) = 98.17, *p* = 0.000, η_*p*_^2^ = 0.75], while the interaction between the groups and the behavior level was significant [*F*(2,33) = 6.73, *p* = 0.004, η_*p*_^2^ = 0.29]. The interaction between the groups and the motion speed was significant [*F*(18,297) = 1.84, *p* = 0.021, η_*p*_^2^ = 0.10], while the rest of the interaction was not significant.

**TABLE 3:** The motion coherence threshold of the youth, the elderly with exercise and the elderly with no exercise group (%, M ± SD).

Speed Group	Youth		Elderly with exercise		Elderly with no exercise	
	70.71%	79.37%	70.71%	79.37%	70.71%	79.37%
1.0°/s	6.70 ± 1.62	12.40 ± 10.01	28.89 ± 29.87	35.21 ± 28.78	34.31 ± 27.98	53.33 ± 24.78
1.4°/s	10.45 ± 11.77	11.94 ± 8.26	21.11 ± 21.88	21.04 ± 15.24	32.01 ± 23.59	42.08 ± 28.24
1.8°/s	6.67 ± 2.53	11.32 ± 8.37	12.64 ± 13.16	16.94 ± 13.92	27.64 ± 23.13	41.49 ± 28.53
2.4°/s	8.58 ± 4.10	10.14 ± 3.33	11.04 ± 5.07	19.83 ± 10.58	28.99 ± 29.05	41.49 ± 25.62
3.0°/s	9.27 ± 3.30	13.61 ± 6.70	13.68 ± 6.24	23.78 ± 14.03	24.24 ± 16.48	41.35 ± 24.28
3.8°/s	12.53 ± 3.99	17.92 ± 15.17	21.81 ± 12.30	31.70 ± 15.44	31.94 ± 17.80	49.38 ± 23.56
4.8°/s	16.11 ± 4.33	21.01 ± 9.08	27.95 ± 17.31	35.56 ± 12.57	42.47 ± 12.39	47.33 ± 14.98
6.0°/s	23.26 ± 10.68	28.82 ± 5.74	44.86 ± 14.01	52.47 ± 13.94	50.14 ± 21.10	63.13 ± 16.74
7.5°/s	34.69 ± 10.83	41.08 ± 12.98	52.99 ± 11.01	62.36 ± 11.24	59.03 ± 18.83	75.07 ± 8.98
9.5°/s	43.75 ± 13.25	57.47 ± 11.07	60.97 ± 14.14	70.45 ± 10.67	68.16 ± 18.85	75.63 ± 10.86

**FIGURE 4:**
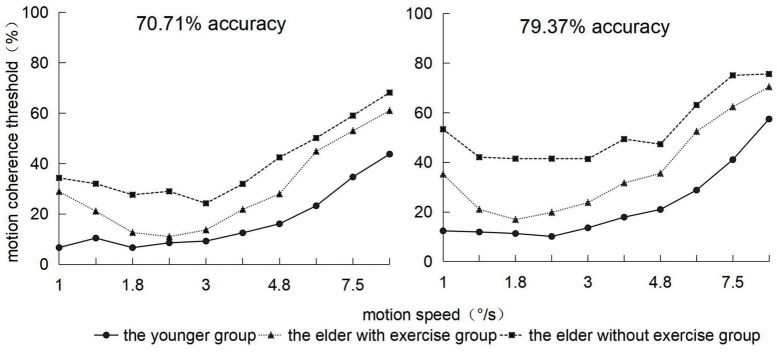
Motion coherence threshold of 3 groups of subjects in the range of 1–9.5 °/s velocity (abscissa) under two behavior levels.

Using simple effect tests to analyze the interaction between the groups and motion speed, we observed that (Figure 4), on the whole, the threshold of the youth group would gradually increase with the speed of point movement, whereas the thresholds of the elderly group with exercise and elderly group with no exercise presented an increasing trend followed by that of decreasing. Under conditions of 1, 6, and 9.5°/s, the threshold of the elderly group with exercise was significantly higher than that of the youth group (*ps* < 0.05), and there was no significant difference with the elderly group with no exercise. Under conditions of 1.4, 1.8, 2.4, 3.0, 3.8, 4.8, and 7.5°/s, the threshold of the elderly group with exercise was significantly lower than that of the elderly group with no exercise (*ps* < 0.05).

#### 3.2.2 The processing mechanism of the aging global kinematic perception

**FIGURE 5:**
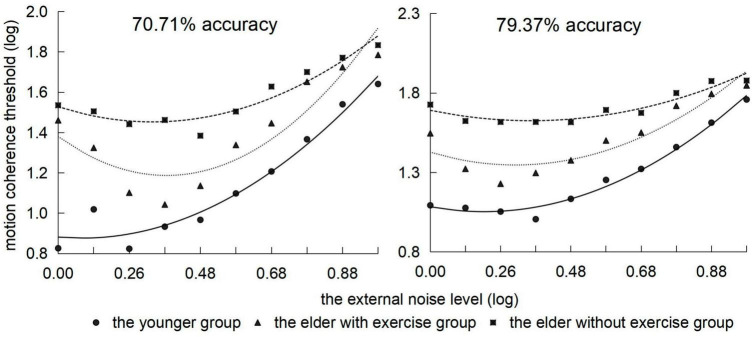
The PTM fitting curve for the 3 groups.

When we compared the fit of each model for the elderly group with exercise, and the elderly group with no exercise, using the *F*-test, we found that (Figure 5; Table 4) there was no significant difference in the fit between the full-parameter model and fitted compact model including *A*_a_ and *A*_f_, but it was significantly higher than other models. We conducted paired-sample *t*-tests to obtain the threshold ratios of the youth group/elderly group with exercise [0.55 ± 0.41 vs. 0.49 ± 0.36, *t*(238) ± 1.18, *p* ± 0.238], and youth group/elderly group with no exercise [0.78 ± 0.76 vs. 0.72 ± 0.54, *t*(238) = 0.71, *p* = 0.476], at two levels of behavior levels. We did not find any significant differences, implying that there was no change in the multiplicative internal noise in the elderly group with exercise, and the elderly group with no exercise, compared to the youth group. Hence, we can see that the compact model including *A*_a_ and *A*_f_ was the best-fitted model (*A*_a_ ≫ *A*_f_) for both the elderly group with regular exercise and elderly group with no regular exercise. This indicated that, regardless of exercise, internal additive noise tended to increase and the ability to eliminate external noise tended to weaken in the elderly groups when compared with the youth group, and the increase in the internal additive noise was even more obvious.

**TABLE 4:** Based-on the youth group, the best-fit model for the elderly groups.

	β	γ	*N*_m_	*N*_a_	*A*_a_	*A*_f_	*A*_m_	*r* ^2^
Youth	0.23	9.87	0.40	1373.30	1	1	1	92.43%
Elderly with exercise	0.23	9.87	0.40	1373.30	2407.63	1.52	1	98.18%
Elderly with no exercise	0.23	9.87	0.40	1373.30	18373.07	1.80	1	98.05%

#### 3.2.3 The impact of exercise on the processing mechanism of GMP in the elderly

Using the threshold of the elderly group with no exercise as the benchmark, we carried out the *F*-test on the fit of each model obtained from the elderly group with exercise and discovered that there was no significant difference between the fit of the full-parameter model and the compact model including *A*_a_ and *A*_f_, but it was more significantly higher than other models (Table 5). We conducted paired-sample *t*-tests to obtain the threshold ratio of the elderly group with exercise/elderly group with no exercise [2.16 ± 2.16 vs. 2.13 ± 1.89, *t*(119) = 0.16, *p* = 0.874], at two levels of behavior levels, and we did not find any significant differences, which indicated that there was no change in the multiplicative internal noise of the elderly group with exercise, when compared with that of the elderly group with no exercise. Hence, the best-fitted model (*A*_a_ < 1, *A*_f_ < 1) for the elderly group with exercise was the compact model including *A*_a_ and *A*_f_, indicating that the internal additive noise decreased and the ability to eliminate external noise improved when compared with the elderly group with no exercise.

**TABLE 5:** Based-on the elderly group with no exercise, the best-fit model for the elderly group with exercise.

	β	γ	*N*_m_	*N*_a_	*A*_a_	*A*_f_	*A*_m_	*r* ^2^
Elderly with no exercise	0.91	1.76	0.65	29.98	1	1	1	85.63%
Elderly with exercise	0.91	1.76	0.65	29.98	0.41	0.93	1	73.08%

## 4 Discussion

This study has found that the threshold of the elderly group with exercise was generally lower than that of the elderly group with no exercise, and the threshold of both groups was higher than that of the youth group. This supports the view of an overall decline in global kinematic perception and also indicated that good exercise habits may have a positive effect on the aging of GMP. At the same time, using the PTM technique, this study also found that the *A*_f_ value of the elderly group with exercise was significantly lower than that of the elderly group with no exercise. This finding indicated that long-term exercise might affect the GMP of the elderly mainly by affecting their ability of eliminating external noise, which provided meaningful guidance and references for improving the aging of GMP in the elderly.

On the whole, this study has found that good exercise habits might have a positive effect on the GMS of the elderly and supports the viewpoint of “the existence of GMP plasticity in the elderly.” In addition, this study also found lower threshold values in the elderly group with exercise in any direction under the horizontal, vertical, rotational, and radial exercise forms. This showed that good exercise habits can universally affect GMP aging. This is could be because of the improvement that exercise has on the brain structure or functions of the elderly. The results of this study are related to those of Shen et al. (2019), who conducted an 8-week Tai Chi (eight forms and five steps) or aerobic exercise (brisk walking) or training on a group of university students and used the MRI scan on the brain structures before and after the training. They found that the gray matter volume had increased significantly in the left precuneus of the aerobic exercise group when compared with the group with no training. For the Tai Chi group, their gray matter volume increased significantly in the left middle occipital gyrus, left precuneus, left superior temporal gyrus, and right middle temporal gyrus. This showed that these two types of exercises could stimulate brain plasticity to different extents. Similarly, Paillard et al. (2015) found that aerobic physical exercises could stimulate neurotrophic factor release and promote angiogenesis, thus promoting the formation of neurons and synapses as well as improving memory and cognitive functions. In Boyke et al.’s (2008) study, the authors observed changes in the gray matter of the aged brain associated with skill association in the hMT/V5 region (middle temporal gyrus region of the visual cortex). A study Colcombe et al. (2006) also found that aerobic exercises could significantly increase the gray and white matter volume in the elderly. GMP-related MRI studies of the elderly revealed that the decreased GMP was associated with reduced local coherence and low-frequency amplitudes in the MT/V5 region in the dorsal visual pathway or with functional changes in the broad brain regions throughout the brain (Jin et al., 2021). The spatial patterns of the gray matter volume signal in the MT/V5 and V3 regions could also effectively predict individual GMP sensitivity (Jin et al., 2020).

From the angle of the cognitive mechanism, this study revealed that the decrease in perceptual efficiency of the elderly during the processing of global motion signals was mainly due to the increase in their internal additive noise and the weakening of their ability to eliminate external noise. Exercise is related to both the change in the GMP sensitivity and changes in the two types of noises. Neurophysiological studies have demonstrated that an underlying source of the increase in internal additive noise among the elderly was due to the increased spontaneous neuronal firing activity with age (Schmolesky et al., 2000; Hua et al., 2006). The decrease in the ability to eliminate external noise was probably due to the decrease in visual attention (Robertson et al., 2013; Shimada, et al., 2013). Previous studies have shown that the differentiation of the overall direction of the random-dot kinematogram paradigm needed to be engaged with the signal-noise ratio during perceptual processing to eliminate disturbances from noise stimuli (Downing, 1988; Hamm et al., 2014). O’Halloran et al. (2014) in their study on the evaluation of the selected responses of 4,315 elderly persons with an average age of 50 found that the speed and accuracy in detecting target stimuli from similar stimuli had decreased significantly in the elderly. A large number of studies have also found that exercise could promote the maintenance of brain functions to a certain extent, and also improve the concentration of the elderly (Chang et al., 2012; Bherer et al., 2013; Muiños and Ballesteros, 2018). For example, Zhang and Gao (2014) in their review of earlier studies found that physical exercise could help to promote the activation of brain regions associated with executive control, thereby maintaining specific cognitive functions. Similarly, Smith et al. (2010) conducted a meta-analysis study on the relationship between aerobic exercise and neurocognitive function spanning from 1966 to 2009 and found that aerobic exercise training could increase the concentration and processing speed, executive function, and memory of an individual. Likewise, Witte et al.’s (2016) study revealed that a 5-month taekwondo training could help to improve the concentration and motor response time of the elderly, while a 10-month training proved to be even more efficient. In like manner, Liu-Ambrose et al. (2010) found that once- or bi-weekly resistance training (such as weightlifting and lunges) over a period of 12 months improved the selective attention and conflict resolution ability in the executive cognitive function of elderly females.

These findings suggested that maintaining good exercise habits might have an indirectly positive effect on the decrease of GMP in the elderly. Among these, relevant factors may include an individual’s brain structure or function, attention and other cognitive factors. However, although we try to control for the influence of several variables, many uncontrolled factors, beyond physical exercise, might have an impact on participants’ performance. For example, Angebrandt et al. (2022) found that even low-level alcohol consumption was associated with premature brain aging. In the study of Linli et al. (2022), using structural magnetic resonance imaging data from the UK Biobank (*n* = 33,293), a brain predictor was trained using a machine learning technique in the non-smoker group (*n* = 14,667), and then tested in the smoker group (*n* = 18,626) to determine the relationships between Brain-Age Gap and smoking parameters. Results showed that smokers showed a larger Brain-Age Gap than non-smokers, more explicitly, the extents vary depending on their smoking characteristic, the increased smoking amount was associated with a larger Brain-Age Gap. They also found that the relationship between smoking and poorer cognition was partially mediated by Brain-Age Gap. Meanwhile, the causal relationship between motion sensitivity and reading skills has been debated for more than 30 years, and Joo et al. (2017) proposed that the correlation between motion processing deficits and reading experience arises from other common mechanisms. From these studies, we are also thinking that since there are so many factors related to individual habits that can affect the aging of GMP, whether the influence of exercise habits on GMP is also mediated by other factors, or whether other factors lead to good overall exercise habits of individuals and thus have an impact on their GMP. As we all know, individuals with good exercise habits may also have other good living habits, such as no smoking, no drinking, stable and happy mood, etc. Therefore, in order to explore the causal relationship between physical exercise and GMP sensitivity in the elderly, the independent variables should be more strictly controlled, or the horizontal and vertical combination method can be used in future studies.

In addition, we didn’t adopted the full 2 × 2 model (young vs. older; with physical exercise vs. without physical exercise) in the two experiments because of the following considerations. Firstly, if the youth group was differentiated, experiment 1 would become a three-factor mixed experimental design (2 × 2 × 8), and experiment 2 would become a four-factor mixed experimental design (2 × 2 × 2 × 10), which would seriously affect the interpretation and understanding of the results. Secondly, the focus of this study is the influence of exercise on the GMP in the elderly. The setting of the youth group was only to prove the existence of aging phenomenon. And, studies on the youth adults so far have found that frequent exposure to dynamic information-rich visual environments could have a positive effect on the GMP, that is, experience-induced perceptual learning, such as badminton, tennis, action video games. Thus, the participants we selected in our study were generally young people with no special sports experience, However, whether the effect of non-fixed form of exercise on global motion perception in the youth adults is also worth studying in the future.

Despite the strength of this study, the following limitations are evident: (1) Ciria et al. (2023) used an umbrella review of meta-analyses to assess the causal relationship between regular physical exercise and the overall enhancement of cognitive function across the lifespan, 24 studies were included. Among these studies, the Cohen’s d varied from 0.23 to 0.36. In our study, we choose a medium effect size as an index to calculate the sample size, which makes the effect size of the result more lower. Although considering the existence of invalid data, we added some participants in our experiment, however, this was not enough to improve the effect size of the results. Thus, this might affect the reliability of this study (Ludyga et al., 2020). (2)The study was unable to eliminate the impact of congenital factors. It adopted a horizontal experimental design and selected individuals with and with no exercise habits; however, the existing differences between the groups may affect the result of the study. Therefore, future research can adopt a longitudinal experimental design and collect larger GMP threshold values from large sample sizes as well as observe the differences between the groups with and with no exercise habits after a period of training. This can then be used to further investigate the effect of exercise on the aging of GMP.

## 5 Conclusion

This study has found a general decline in GMP across exercise forms and directions in the elderly; however, maintaining at least 7 h of exercise every week might have a certain degree of positive effect on GMP aging. And this possible effect in GMP through exercise might be mainly due to the attenuation of the individual’s internal additive noise and an improvement in the ability to exclude external noise.

## Data availability statement

The raw data supporting the conclusions of this article will be made available by the authors, without undue reservation.

## Ethics statement

The studies involving humans were approved by the Ethics Committee of Mental Health Education Center of Zhengzhou University. The studies were conducted in accordance with the local legislation and institutional requirements. The participants provided their written informed consent to participate in this study.

## Author contributions

ZL: Conceptualization, Data curation, Formal analysis, Writing—original draft. LZ: Data curation, Project administration, Visualization, Writing—review and editing. PW: Data curation, Writing—original draft. YZ: Formal analysis, Writing—original draft. YX: Formal analysis, Writing—original draft. HJ: Funding acquisition, Writing—review and editing.

## Footnotes

1
https://www.mathworks.com/
